# Glasgow Royal Infirmary
*Glasgow Journal, No. XIII.


**Published:** 1831

**Authors:** 


**GLASGOW ROYAL INFIRMARY.***

TRAUMATIC TETANUS.

Two cases of this terrible disease are recorded, with the view of adding something to our stock of information, if such it can be called, respecting its pathology. We shall notice them very shortly.

*Case* 1. Patrick Vailly, æt. 15, was blown in the air and a good deal scorched, on the 17th April, 1830, by the explosion of a steam-engine boiler. Some collapse succeeded the injury, and after that pain in the abdomen requiring depletion. On the 27th he was convalescent, when he complained of pain in the abdomen, not increased on pressure, and on the 29th there was opisthotonos. On the 3d of May the patient died. The treatment consisted in purging with calomel and castor oil and blistering the spine. Immediately after death the body was placed with the face to the floor.

*Sectio Cadaveris*:—24 *hor. post mortem.* "The whole spinous processes and calvarium were removed, the brain and thecæ vertebrarum fully exposed. There was a little serous fluid at the base of the brain, betwixt the tunica arachnoidea and pia mater. The brain was considerably more vascular than usual, and on the posterior part of both lobes of the cerebellum there existed an ecchymosed appearance, which could easily be removed by raising the pia mater. The medulla spinalis had a perfectly healthy appearance, but a considerable quantity of partly fluid, partly coagulated blood, existed betwixt the theca and the vertebræ. The vesicated surfaces occupied the lower half of the left leg, and the outer and lower half of the right leg. Both had a green sloughy aspect, and the cellular substance was much inflamed. The veins did not appear more vascular than natural, and the arteries appeared healthy. The nerves were also carefully examined; the cutaneous of both legs, particularly the communicans tibialis and the communicating branches of the peroneal nerve with the tibialis communis, were inflamed at the seat of the injury; tracing them upwards above this point they were perfectly healthy, except that portion of the peroneal which turns over the head of the fibula, there it was again distinctly very vascular, thus leaving an intermediate portion perfectly free from the appearances of inflammation. The vascularity appeared to be confined to the sheath of each nerve; the deep-seated branches appeared to be quite natural. No other morbid appearances were detected."

*Case* 2. William Fleming, æt. 17, had the ring and middle fingers of the right hand much lacerated and injured by machinery on the 14th July, 1830; the last phalanx of the middle finger was only adherent to the second by a small slip of skin, and it was removed accordingly. On the 21st he began to experience severe pain in the fingers, and at the same time he was seized with tetanic symptoms He was admitted on the 22d. The tetanic symptoms were somewhat aggravated, and

* Glasgow Journal, No. XIII.

the second and last phalanges of the injured fingers were completely gangrenous, and the second phalanx of both was found fractured. The fingers were removed, and calomel, with leeches to the nucha, and purgatives employed. We need not mention the subsequent symptoms or treatment, suffice it that the patient died early on the 24th, the spasms continuing both frequent and severe.

*Sectio Cadaveris* :—24 *hor. post mortem.* "The body was allowed to lie the usual way on the back till the time of inspection. The calvarium and spinous ridges were removed, fully exposing the theca vertebrarum, down to the cauda equina; there was no effusion on the brain or its membranes, and its substance was natural throughout. No effusion existed between the theca and the vertebræ; the theca was healthy, and betwixt it and the spinal cord was a preternatural quantity of serum. The cord itself was of a pale colour. The nerves on each side of the remaining phalanx of the ring finger were very vascular. On tracing upwards the ulnar nerve from this point to the elbow, it was of its natural colour, but here again it became very vascular for about the extent of two inches. In the axilla it again presented a similar appearance as at the elbow, the portion of it intervening betwixt these two points being healthy. Tracing the median nerve in the same way as the ulnar, it was found perfectly natural, from its digital branch, which supplied the radial side of the ring finger, (and which, as stated above, was much inflamed,) till about the middle of the arm, when it again presented an inflamed appearance for the extent of one and a half inch. The portion of it intervening betwixt this part and that confined to the axilla, where it again became vascular, was natural. This vascularity throughout, was not confined to the sheaths of the nerves, but occupied their substance; the radial and superficial nerves, of the arm, along with its veins and arteries, were perfectly natural; the lumbar nerves were unaffected; the œsophagus was examined, and found healthy; the trachea appeared inflamed, and contained a large quantity of greenish coloured mucus; the other thoracic viscera and digestive organs natural."

The inference which appears to be drawn from the foregoing case by Dr. Perry, is, that he would be warranted in treating any case of the kind, as a local inflammation of the nerves leading from the seat of the injury, the interruption of the suppurative process in the wound being one of the first appearances. We should be sorry to form these or any other conclusions on two such cases.

In a case of lithotomy related by Dr. Perry, nine stones were found in and removed from the bladder. The prostate was enlarged; the symptoms of stone in the bladder had existed for ten months previous to the performance of the operation. Two of the stones were situated in the inferior fundus of the bladder, and were of a flattened shape. The remaining seven were placed at the upper part of the bladder behind the pubes, each of the size and figure of chesnuts, and lying as if they had all been compressed and wedged together into the form of a ball. The stones were composed of the triple phosphate with a nucleus of lithic acid. The urine had occasionally contained "sand and slime," but never any blood.

### SUDDEN DEATH FROM DREAD OF OPERATION.

A man aged 40, was admitted with very severe laceration of the left fore-arm, which had happened on the preceding day from the limb being drawn between two wheels. The integuments above the wrist-joint were emphysematous, the wounds emitted a gangrenous fetor, the pulse could not be felt at the wrist, the tongue was white, the skin warm. Amputation was proposed and rejected; a tourniquet was kept constantly round the arm, and port wine poultices applied.

The case went on well for six or seven days, when the line of separation between the sound and gangrenous parts was distinct. The injury done to the hard and soft textures of the fore-arm was too great notwithstanding to admit of any reasonable expectation of being enabled to save the limb, and a communication to that effect was made in a forcible manner to the patient. His hopes had been previously excited—they were now damped—he began to shake—and earnestly declared that he could never submit to the operation. He soon afterwards sank into a comatose state—complained of nothing—the pulse was weak—the pupils were natural — a cold sweat over-spread the body — ammonia and brandy were exhibited, but he sank, and died at 1, p.m. next day. On dissection, a tea-spoonful of serum was found in both ventricles. The radial, ulnar, and anterior interosseous arteries of the injured fore-arm were ruptured; the bones were denuded of their periosteum and rasped.

We cannot but imagine that surgeons are often not sufficiently careful, in hospital practice, of informing patients that they must submit to operations. On one or two occasions we have seen bad effects from breaking intelligence of this kind too abruptly. We have seen a small wound in the thumb assume a sloughing character, immediately after a communication which alarmed the patient in a considerable degree.

### Aneurism by Anastomosis.

*Case* 1. A girl, æt. mens. 18, admitted May 1st, with a large tumour, of a purple cast and doughy feel, occupying the radial half of the palm, and part of the ball of the thumb of the left hand; it frequently discharged a considerable quantity of blood through three small openings on its surface. Pressure diminished the tumour much, but its size returned on its withdrawal. Some months previously its removal by the knife had been attempted; it had been twice punctured, and some difficulty arose in arresting the bleeding. On the 4th May the tumour was removed by incision, and the actual cautery afterwards applied. On the 21st the patient was dismissed, with the wound nearly cicatrized.

*Case* 2. A child, aged seven months, admitted with a tumour an inch long and half an inch broad at the root of the nose, of purple colour, with small red vessels on its surface, reducible by pressure to a third of its dimensions. It occupied the whole space between the inner canthus of the right eye and the mesial line of the nose, and extended from the inner end of the right eyebrow to an horizontal line drawn across the face from the right ala of the nostril, covering the lachrymal duct and a third of the lower eyelid. When partially emptied, its attachment to the subjacent parts seemed firm at the inner angle of the eye only. Three months previously the child had received a blow upon the part from the edge of a hat; in a week afterwards a small purple spot was observed, which had since continued to increase.

For three weeks pressure on the tumour was made by a spring and pad, under which it ulcerated deeply, but again increased to its former size, on the withdrawal of the pad. An attempt to destroy it with the caustic potash failed next; and this was succeeded by the actual cautery, which seemed to accelerate the extension of the tumour. An incision was made at the lower portion of tumour, and carried along the side of the ala-nasi to its upper part at the end of the eyebrow; and an attempt made to extirpate it from below. The flow of blood was, however, so rapid and profuse, that to prevent fatal consequences, it was necessary to desist, and apply the actual cautery, which was in readiness. The bleeding was by this means stopped, and the wound dressed with resinous ointment. On the following day, the child was excessively weak, and refused the breast. It had been ordered a little wine after the operation, which was occasionally given for the next two days. By the 25th the tumour had greatly diminished, though still a little puffiness remained on the nasal edge of the upper eyelid, which was occasionally touched with the argent. nitrat. The mother wishing to go home, was desired to return in a fortnight, which she did. Nothing farther was done; the tumour gradually contracted; the skin is natural in colour; the eye is perfect. With the exception of a little contraction of the internal angle of the upper eyelid; the cicatrix is scarcely perceptible.

We may here introduce a proposition of our friend Dr. Marshall Hall's, which appears in a late number of the Medical Gazette. In a case of oval nævus, rather larger than a shilling, which the Doctor attended with Mr. Heming of Kentish Town, he adopted the following plan. A couching-needle with cutting edges was introduced at one point of the circumference of the nævus, close by the adjoining healthy skin, and thence was made to pass through the tumour in eight or nine different directions, so as to produce slight incisions through its textures, parallel with the skin, but not so as to pierce the tumour in any other part. A little pressure by means of strips of adhesive plaster was applied over the tumour. For weeks no apparent effect was produced, but in half a year the tumour was found to have disappeared, and the colour of the skin to be nearly natural, without any appearance of scar. We hardly know what credit to attribute to the operation, for the cure was very tardy. The object of Dr. Hall is to prevent scar. The operation might be repeated at longer or shorter intervals, and with more or less numerous punctures, according to circumstances. But to return to Dr. Perry's report. He appears to be an advocate for the torsion of arteries now extensively practised by M. Amussat.

"I have generally applied ligatures to the large arteries, treating the smaller by torsion. In the fingers and toes torsion only was used, and in the mammæ, generally torsion; in the latter cases, and tumours, it is particularly useful, as it is often necessary to dissect deeply under the skin, and the leaving of ligatures keeps up a purulent discharge; when ligatures are used in such cases, it is better to use small silk ones, and cut away both ends. The practice of suppressing hæmorrhage by torsion, or bruising of the arteries, is not altogether new; I partially practised it in 1820, along with my then colleague, the late Dr. G. C. Monteath. The circumstance which gave rise to it, was a casual conversation which occurred respecting a case of laceration of the arm by machinery, where the vessels were torn across, and no bleeding followed, a circumstance by no means unfrequent. It was a natural inference, that by bruising the arteries, destroying their vitality for a short space, they would soon cease to bleed, as a coagulum would form in their inactive extremities. From this analogy, we frequently had recourse to bruising or twisting the smaller arteries during the operation, without thinking it a matter of such importance as to call it a discovery. I do not mean by this to detract from the merit of M. Amussat, who has recommended and carried the practice farther than I have yet ventured to go. As it is often of great importance during operations to prevent as much as possible the loss of blood, whether arterial or venous, I have been in the practice of using a pair of button forceps for laying hold of the bleeding vessels, and twisting them, if arteries, or if large veins, allowing them to remain fixed till the operation is finished, and the tourniquet removed. In this way much blood may be saved, and little time lost, and in removing tumours, it prevents the operator from being embarrassed with the blood.

The button forceps may also be applied to an equally, if not more important, class of cases; I mean, for the cure of recently-punctured arteries, or in place of ligatures in cases of aneurism. If a wound is made, for instance, in the brachial artery, by bleeding, the external wound may be enlarged, and the side of the artery, where the wound is, grasped by the forceps, the button pushed down, and the forceps left for 24 hours in the wound, (the adhesion will take place in less time,) firmly fixed by means of the head of the forceps being passed through a slit in a piece of sponge, and this again fixed on the arm, to prevent motion, by strips of adhesive plaster. The forceps must be small, and rounded at the point, (perhaps those made of silver would suit best, as they do not corrode,) and the wound closed and kept together on each side of the forceps by strips of plaster, or, if it is wished, the whole caliber of the artery may be included in the forceps, and the flow of blood completely interrupted, till the sides of the vessel adhere. I may be asked, what advantage this has over the ligature? In the first place, it is easier applied, there is less danger of including the nerves, there is no risk of insulating the artery from its connexions, and, consequently, less of secondary hæmorrhage. I shall, however, postpone any farther particulars respecting this plan of treatment until the next Number of the Journal."

We cannot say that the foregoing statements bring conviction to our minds. We have been and continue to be sceptical respecting the advantages, nay, even the safety of M. Amussat's method. We would not oppose a plan because it is new, but neither would we adopt it because it is new. It requires both caution and time to appreciate the merits or the mischiefs of all new modes of practice. The advocates for torsion ground their arguments on the fact that lacerated arteries frequently do not bleed. Very true; but occasionally they do so, and not very unfrequently secondary hæmorrhage occurs from them. However, as we have already hinted, we yet want many facts to justify a sound and practical man in assenting to M. Amussat's opinions. The statements of M. Amussat himself are certainly strong, but what new medicine or what new operation ever wanted powerful *facts* to support it? On the 31st January M. Amussat introduced to the Academy of France four patients on whom he had operated. Three were children of seven, nine, and twelve years of age, who had had their right thighs amputated about the same time, for white-swelling of the knee-joint. The next was a man fifty years of age, the end of whose humerus was shattered by a ball; in him the amputation was not performed till the 26th day. The arteries were twisted in all, and secondary hæmorrhage occurred in none. In the youngest patient the wound was perfectly healed by the first intention in seven days. We repeat that evidence enough has been brought forward by M. Amussat to prove that his proposal is not to be scouted: but not enough to induce good surgeons to yield blind acquiescence and assent to all the advantages claimed for it by its author. Time, and time only, will determine the merits of the case.

---

